# Effective publication strategies in clinical research

**DOI:** 10.1371/journal.pone.0228438

**Published:** 2020-01-30

**Authors:** Daniella B. Deutz, Evgenios Vlachos, Dorte Drongstrup, Bertil F. Dorch, Charlotte Wien

**Affiliations:** University Library, University of Southern Denmark, Odense, Denmark; KU Leuven, BELGIUM

## Abstract

Researchers in Europe are increasingly assessed by their publication metrics. To uncover the effect of quantitative assessment on the publication strategies of clinical researchers in Denmark, we interviewed 9 senior researchers at the Department of Clinical Research at the University of Southern Denmark with the lowest and highest values for *a*, as defined by Hirsch. Our aim is to investigate the importance of these metrics to their academic careers: h-index, number of publications, number of citations, international collaborations, local collaborations, field specific journal publishing and high journal impact factor publishing. To validate our findings we compared their publication record to their statistically analyzed stated publication strategy. Our results indicate two styles of publication strategy used by these senior researchers. Researchers with Low *a* engage in local collaborations, disseminate knowledge in local media and publish in field specific journals, while researchers with High *a* engage in international collaborations, invest significant time in publishing in the highest impact journals in their field, and acquire a greater number of citations. Both publication strategies can lead to a successful academic career, yet we have an indication through the h5-index that the practices of the High *a* group are more likely to nudge the h-index.

## Introduction

Researchers in Europe are occupied with a wide range of tasks outside of research, such as teaching, managing projects, curating data, and writing funding applications to name a few, which limits their time to perform research and write publications. Ideally, when it comes to scholarly communication, one’s focus should be on the dissemination of knowledge. One should aim to publish only the most definitive work, of highest quality, in the most reputable journals in the field. Yet, when researchers are judged by the absolute value of their publication metrics, conflicting incentives come into play [1]. Research decisions may be influenced by the effect on a researcher’s publication metrics, rather than by scientific merit [2]. A researcher could be working in a trending field due to personal interest, ease of funding, or because they believe their publications are more likely to get into high impact journals [3]. Similarly, a researcher could be writing guidelines and reviews, citing their own work several times in each publication because the science merits it, or simply to increase their number of citations [4–7]. One could share new publications on social media to reach new readers, or simply to boost their Altmetric scores. One could seek out international collaborators to garner fresh insights and generate new questions, or simply to add their name to a growing list of co-authors to improve access to “better” journals and a broader network.

Researchers are aware that their publication metrics are being used in tenure track assessments, grant applications and job interviews and target them accordingly [8–14]. Any metric that can be measured, can be gamed and naturally, there are many methods in use whose principle aim is to boost the h-index, *h*, [15,16] number of publications, *N*_*P*_, [17,18] and number of citations, *N*_*C*_ [19,20]. All the while every year there are new proposals for how a researcher’s impact should be measured [21–27].

We asked successful researchers how they have planned their publications and deal with these conflicting incentives in academic publishing, when the measurement methods are constantly changing. Researchers were asked about their publication strategies, a recognized term in Scientometrics, which Joubert & Rogers (2015) [28] describe as: “*a road map delineating what*, *when*, *and how research will be published*. *It should include the type of article and journal*, *based on the target audience*”. In addition, if they do focus on particular metrics, they were asked to report them. We define a successful researcher as someone who is an established researcher in their field with a permanent position at their institute, and an effective publication strategy as one that allowed them to achieve such a position. We focus on the h-index, *h*, as it is widely used to rank academics [29–32] and group researchers by the degree of efficiency, *a*, where *a = N*_*C*_*/h*^*2*^ as taken from Hirsch 2005 [29], and support a qualitative investigation of the publishing practices of a group of clinical researchers, based on semi-structured interviews, with a quantitative investigation of their publication metrics. Our main aim is to examine the behavior of high *a* and low *a* researchers in relation to what role the importance of a high h-index, number of publications, number of citations, international or local collaborations, field specific journal publishing or high journal impact factor (JIF) journal publishing plays in their career. Researchers in clinical science are in a special position, as their discipline offers the potential to publish in journals of the highest JIF of any field. We therefore assume that if optimizing behavior occurs, we will find it in this field.

In the following sections we explain how we sampled researchers to interview and present the analysis of their interview responses. For validation purposes, we then compare the researcher’s stated publication strategies with their publication metrics from the last 6 years and discuss the implications.

## Methods

### Selection of interviewees

We invited 18 researchers to take part in a recorded interview regarding their h-index and publication metrics based on the degree of efficiency, *a*, where *a = N*_*C*_*/h*^*2*^, of their publications in Scopus. The *a* was chosen over the h-index, as the h-index is a cumulative metric, where higher values likely indicate older researchers, while the *a* could provide more immediate insight into their publication strategies. At first glance, the degree of efficiency, *a*, would appear to have no correlation with the h-index (r = 0.07, S1 File). Yet, the *a* is indicating a flaw in the h-index, where highly cited articles cease to impact the metric. Researchers with a high value for *a* could have only a few extremely well-cited publications or have well cited publications overall. While researchers with a low value for *a* could be in a field where citations are not so freely given, they could have well cited citations overall, or they could simply be poorly cited. To fully understand the data, input from the actual researchers is needed. At 71%, the bulk of the researchers have a degree of efficiency, *a*, clustered between 3 < *a* ≤ 5, with an average *h* of 33±16. This is similar to the spread reported by Hirsch 2005 [22] for physicists. To uncover differences in the researcher’s publication strategies, we selected researchers to interview from the outlying groups with a degree of efficiency, *a*, below 3 (n = 10) and above 5 (n = 8). All invited researchers are senior faculty, have PhD students under their supervision and have an extensive network.

In total, we conducted 9 interviews (5 with *a* ≤ 3 and 4 with *a* > 5). The remaining 9 invitees did not respond or declined our invitation. The interviews were semi-structured, allowing room for openness and broader conversation around the main interview framework which can be found in the S1 File. We preferred this method over an online survey or a questionnaire in order to elicit more spontaneous, and perhaps more truthful, responses as the interviewee is allowed to talk freely. Each interview lasted approximately 10 minutes and was conducted in the office of the interviewees. Interviewees gave verbal consent for being recorded and participated freely and voluntarily in our study. To minimize the time investment for the interviewees, verbal consent was recorded at the start of each interview in lieu of written consent.

### Interview analysis and publication metrics

Transcriptions of the audio recordings were separately scanned for meaning by three of the authors. To perform a statistical analysis and compare stated publication strategies to actual publication outcomes the responses of the researchers were independently distilled into simplified yes or no answers by three of the authors. Elaborations on their answers and quotes are included in the discussion. Responses from the interviewees were analyzed using multiple correspondence analysis (MCA), commonly used to analyze categorical survey data [33]. We used the FactoMineR [34] and factoextra [35] packages in the R Statistical Computing software to run the analysis. The responses were compared to their actual behavior by tracking their publication metrics from the previous 6 years (2013–2018) in SciVal. In addition, we generated a list of all the Scopus sources (journals indexed in Scopus) that the authors published in and compiled two lists for the two types of interviewees. We then counted how many times each group published in the top 30 JIF journals, ranked by Journal Citation Reports (JCR), for medicine on Feb 14^th^ 2019 from 2013 to 2018. We complied with the terms of services for collecting data from Scopus, SciVal and JCR.

## Results and discussion

### Interview analysis

The interviewed researchers were asked about what they took into consideration when planning their publications (see the S1 File for the interview guide). What did they consider before submitting an article to a journal or conference, for instance did they look up the journal impact factor (JIF)? Did they generally work with international collaborators or local ones? To get a sense of their awareness of their publication metrics, they were asked about the importance of their number of publications, number of citations, and h-index to their work. Their responses are summarized in Table 1, where each researcher’s responses are simplified to indicate whether they find each publication metric (or variable) important to their work (yes) or not (no).

**Table 1:** Responses of interviewed researchers to what publication metrics they find important to their work. The degree of efficiency, *a*, separates the researchers into “low a” and “high a”. The remaining 7 variables categorize the researchers into those who place importance to a variable (yes) and those who do not (no). The 7 variables are: *N*_*P*_ (Number of publications) importance, *N*_*C*_ (Number of citations) importance, h-index importance, high JIF importance—importance of publishing in high impact factor journals, field specific importance–importance of publishing in field specific journals, Local collaboration importance, and International collaboration importance.

Researcher	*a*	*N*_*P*_ importance	*N*_*C*_ importance	h-index importance	high JIF importance	field specific importance	local collaboration importance	international collaboration importance
1	Low	Yes	Yes	Yes	No	Yes	Yes	No
2	Low	Yes	No	Yes	No	Yes	Yes	Yes
3	Low	Yes	No	Yes	No	Yes	Yes	Yes
4	Low	Yes	Yes	Yes	No	Yes	Yes	Yes
5	Low	Yes	No	No	No	Yes	Yes	Yes
6	High	No	Yes	Yes	Yes	No	Yes	Yes
7	High	Yes	Yes	No	Yes	No	Yes	Yes
8	High	No	Yes	No	Yes	No	No	Yes
9	High	No	Yes	No	No	Yes	No	Yes

**Fig 1:**
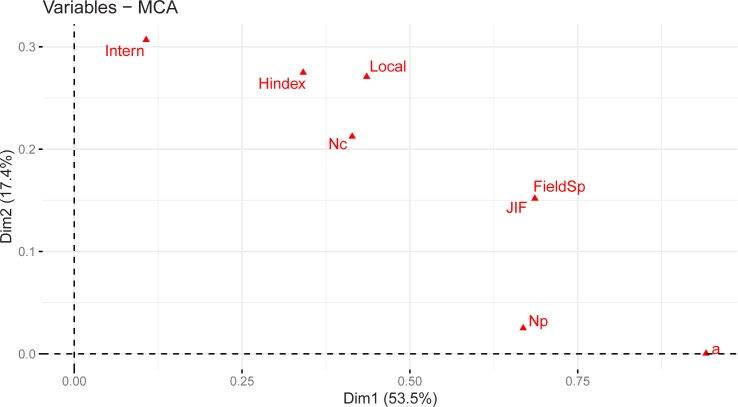
MCA of publication metric variables. Correlation of each variable from Table 1 to the best linear combination of the variables so that the variance along the new dimension is maximum, given here by Dimension 1 (Dim1) and Dimension 2 (Dim2). The square of the correlations between the variables and the dimensions are used as the coordinates of each variable.

We used multiple correspondence analysis (MCA) to identify potential associations between the importance of each publication metric and to identify groups of individuals with similar profiles in their answers. Table 1 is used as input for the MCA. The correlation of each of the 7 variables from Table 1 to the two dimensions of the MCA is shown in Fig 1, where the two axes represent the best linear combination of the variables so that the variance along the new dimension is maximum. The two dimensions are sufficient to retain 71% of the total inertia (variation) contained in the data. Not all the variables are equally well displayed in the two dimensions. The variables *a*, *N*_*P*_, high JIF, and FieldSpecific are more correlated with Dimension 1, while the variables h-index, international collaboration and local collaboration are more correlated with Dimension 2. It is clearly shown that the variable *a* is contributing heavily on Dimension 1 and is highly correlated with it.

**Fig 2:**
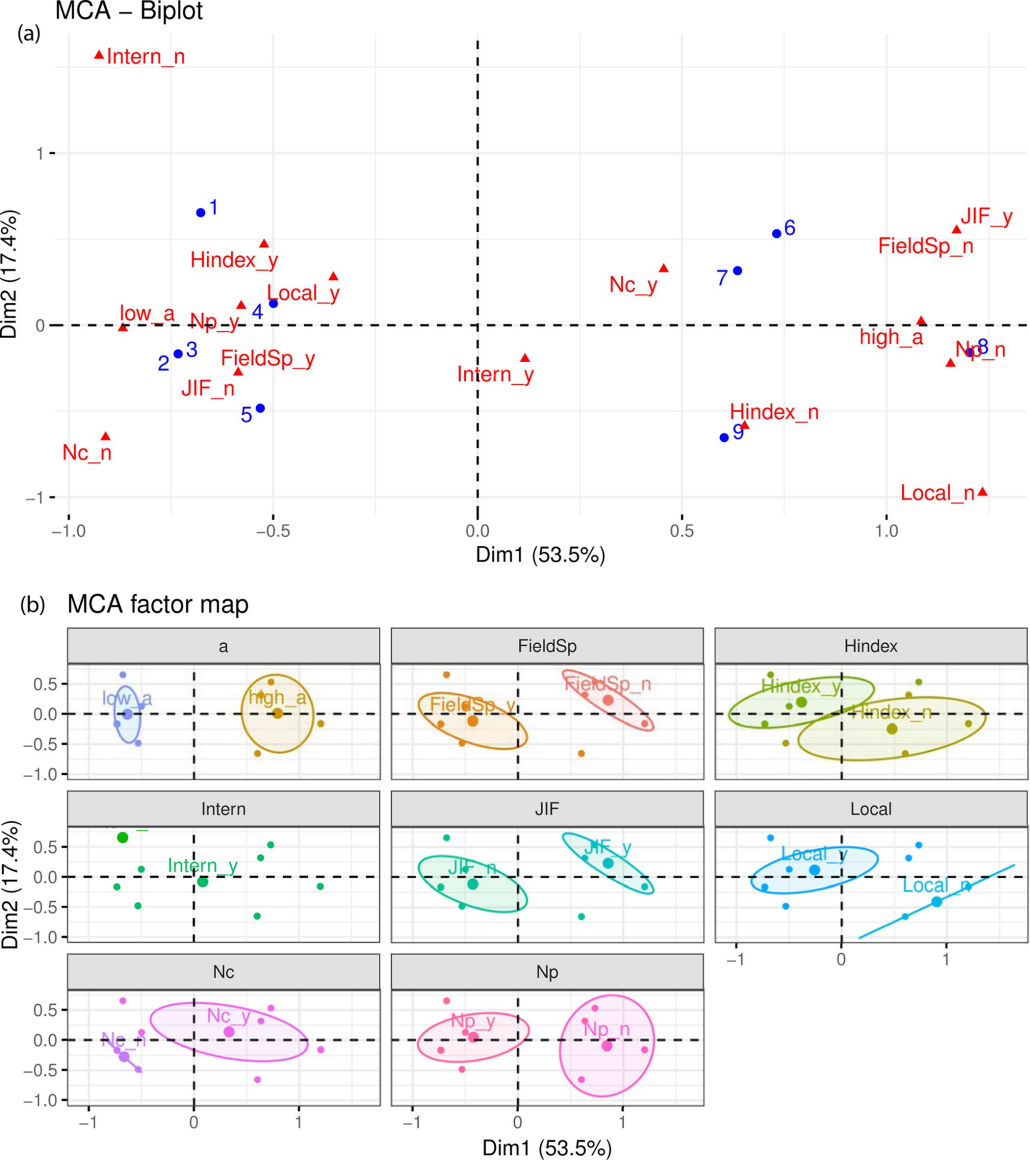
MCA positioning of individual researchers around the publication metric variables. (a) MCA biplot positioning the researchers and variables with respect to the two dimensions. The blue circles indicate the researchers. The red triangles indicate the variables, where the 7 original variables are split into the position for a “yes” or “no” answer by the sub-indices “_y” and “_n” respectively. (b) MCA factor map grouping the interviewed researchers per publication metric variable, shown in a different color for each variable. The ellipses indicate potential groupings of the researchers by their response of whether each publication metric was of importance to them or not.

To identify global patterns within the data, the correlation of each researcher (represented by the blue circles) and each variable (represented by the red triangles) to the two dimensions is shown in the MCA biplot (Fig 2A). The distance between each researcher and variable shows how similar or different they are to one another, where similar points are closer together and dissimilar points are farther apart. Two broad clusters, grouping researchers with either a High or Low *a*, can be distinguished based on their positive or negative correlation with Dimension 1. Overall, researchers with High *a* (numbered 6–9) place importance in high JIF journals, the number of citations, and international collaborations while they do not place importance in field specific journals, the h-index, local collaborations or the number of publications. On the other hand, researchers with Low *a* (numbered 1–5) place importance in field specific journals, the h-index, international and local collaborations, and the number of publications, while they do not place importance in high JIF or the number of citations. From the centrality of the variable in the factor map it can be inferred that international collaborations are important to all researchers.

A more detailed view of this clustering of researchers is shown in Fig 2B. Here we see that for each publication metric but international collaborations, there is a clear divide between researchers with a High *a* or Low *a* based on their positive or negative correlation with Dimension 1. The greater axis of each ellipse skews towards Dimension 1, indicating that it is the most influential dimension.

### Actual publication metrics

To examine whether the publication metrics the interviewed researchers indicated as important to their publication strategies are influenced at all by their actual actions and practices, a quantitative analysis of their publications from 2013 to 2018 was performed. To indicate focus on *N*_*P*_, *N*_*C*_, h-index, high JIF, field specific journals, local collaborations and international ones, we examined the publication metrics given in Table 2.

**Table 2:** Actual publication metrics used to examine preference for each of the initial publication metric variables in the researcher’s publication strategies.

Initial variable	Symbol	Actual publication metric
*N*_*P*_ importance	*N*_*P6*_	Total number of publications published from 2013–2018
*N*_*C*_ importance	*N*_*C6*_	Field-Weighted Citation Impact of *N*_*P6*_
h-index importance	*h5*-index	the h-index only taking into account the previous 5 years
high JIF importance	*JIF*_*6*_	Percentage of *N*_*P6*_ in the top 1% of journals by their Scimago Journal Ranking or SJR
field specific importance	*Field*_*6*_	Percentage of *N*_*P6*_ in the bottom 90% of journals by their SJR
local collaboration importance	*Local*_*6*_	Percentage of *N*_*P6*_ with national collaborators, and without international collaborators
international collaboration importance	*International*_*6*_	Percentage of *N*_*P6*_ with international collaborators

In Table 3 the value of these actual publication metrics is listed, for each researcher. The variables are used to broadly support each researcher’s stated focus on *N*_*P*_, *N*_*C*_, h-index, high JIF journals, field specific journals, local collaboration and international collaboration, respectively. Within this time period, researchers with High *a* have an overall higher average *N*_*C6*_, *h5*-index, and international collaboration rate, while researchers with Low *a* have published a larger percentage of their publications in lower ranked journals (*Field*_*6*_). The average number of publications and percentage of local collaborations is almost the same for High and Low *a* researchers, while the percentage of publications in the top 1% of journals (*JIF*_*6*_) is highly researcher dependent.

**Table 3:** Actual publication metrics of interviewed researchers based on publications from 2013–3018 used as supplementary variables for the MCA. The degree of efficiency, *a*, separates the researchers into “Low *a*” and “High *a*”. The remaining 7 variables, as explained in Table 2, can indicate each researcher’s focus on *N*_*P*_, *N*_*C*_, h-index, high JIF journals, field specific journals, local collaboration and international collaboration, respectively.

Researcher	*a*	*N*_*P6*_ [–]	*N*_*C6*_ [–]	*h5*-index [–]	*JIF*_*6*_ [%]	*Field*_*6*_ [%]	*Local*_*6*_ [%]	*International*_*6*_ [%]
1	Low	28	1.5	9	0	79	18	18
2	Low	96	1.5	15	3	50	35	36
3	Low	17	2.1	6	0	47	24	35
4	Low	39	0.8	8	0	56	33	13
5	Low	47	1.3	6	4	57	21	17
6	High	32	9.5	13	44	25	31	50
7	High	62	1.7	11	0	39	6	58
8	High	39	7.1	13	0	21	15	54
9	High	63	2.0	11	8	54	33	59

For this supplementary set of quantitative variables from Table 3, we proceed with a similar MCA. We assume that the higher the value of the supplementary quantitative variable from Table 3, the closer this will correspond to the variable being of importance to the researcher from Table 1. For instance, if a researcher indicates an importance for the number of publications during the interview, that will correspond to an actual high number of publications over the last 6 years.

**Fig 3:**
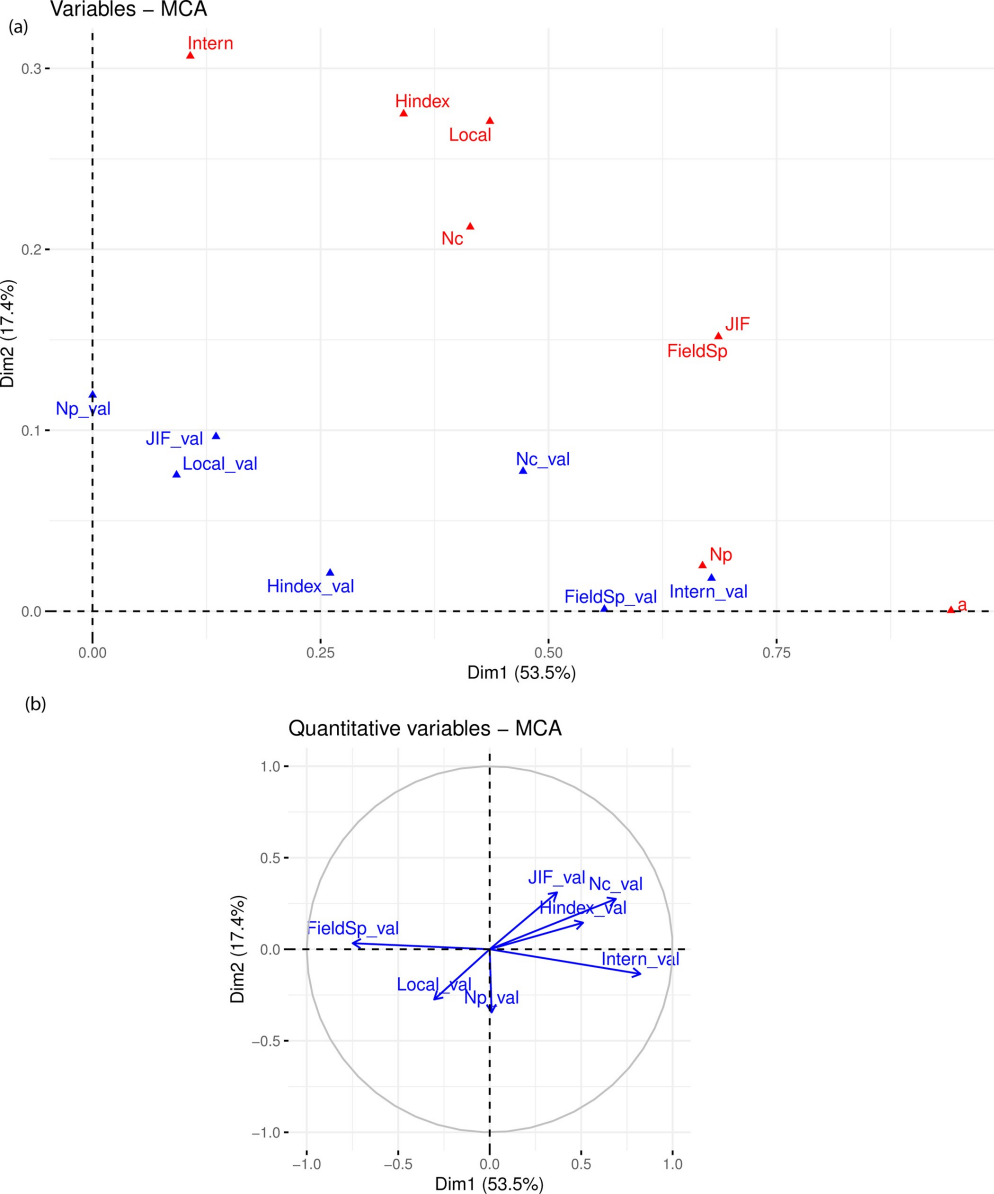
Multiple Correspondence Analysis (MCA) of researcher responses compared to their actual publication metrics. (a) Correlation of the reviewer responses, indicated by the red triangles, and actual publication metrics from the last 6 years, indicated by the blue triangles and “_val” sub-indices, to the two dimensions. (b) Correlation circle representation of the actual publication metrics in the two dimensions, where each variable is represented by its correlation coefficience with the dimension.

The results of the MCA based on both the researcher’s responses, and their publication metrics are shown in Fig 3A. Overall, the actual publication metrics of the researchers (indicated by the blue triangles and “_val” sub-indices) are spread in the same global pattern as their responses (indicated by the red triangles). The actual publication metrics are less correlated with Dimension 2 than the researcher responses, as they appear lower in Fig 3A. In Dimension 1 we can see that the position of the *N*_*C*_, h-index, and perhaps the field specific journal variables is almost aligned with their respective supplementary variables, and in Dimension 2 the same applies to the high JIF variable.

As the actual publication metrics are quantitative, to compare the positioning of these supplementary variables to the researcher responses we show the correlation circle representation in Fig 3B instead of a biplot. The variables for local collaborations and field specific journals are negatively correlated with Dimension 1, positioned in the Low *a* plane. High JIF journals, international collaborations, *Nc* and the h-index are positively correlated with Dimension 1, positioned in the High *a* plane, and only the number of publications is not correlated with Dimension 1.

### Discussion of researcher preference, publication metrics and interview analysis

Our results indicate that there are two publication strategies in use by researchers, which can be distinguished based on their degree of efficiency, *a*. Researchers with a Low *a* prefer to publish in field specific journals, often resulting in a journal with a lower journal impact factor (JIF) and SCImago journal rank (SJR). In their responses they indicated they prioritize the number of their publications, *N*_*P*_, focusing on the topic and readership. Yet, this focus did not translate to a higher *N*_*P6*_ (Table 3) in the 6 years we examined. They “w*ant the message out where it is understood*” as one of our interviewees formulated it, and often appear in the public arena, such as national media.

Researchers with a High *a* indicated they prefer to publish in journals with the highest JIF, and this is largely corroborated by their lower overall publication rate in the bottom 90% of journals ranked by SJR. They prioritize their number of citations, *N*_*C*_, and did indeed receive a higher rate of citations than would be expected for the average publication in their field, as signified by the *N*_*C6*_. Even though they claim not to focus on their number of publications, there is no measurable difference between the publication rate of the two groups. Indeed, from the MCA Fig 3B it is clear *N*_*P*_ is not dependent on Dimension 1, and therefore probably not dependent on *a*. Furthermore, they do indeed focus on establishing international collaborations, publishing 55% of their articles with international collaborators as opposed to the 24% of the researchers with Low *a*. As one of the researchers put it, they believe that “*collaborating is how you learn to do good research*” and select international collaborators based on their potential.

Interestingly, researchers with High *a* had a higher h5-index (Table 3) than those with Low *a* even though they indicated that the h-index was of no importance to their publication strategies. This inversion between publication metric preference and actual publication outcome is supported by the results from the MCA, where the h-index is negatively correlated with Dimension 1 in Fig 2A and positively correlated in Fig 3B. In their interviews, researchers with Low *a* were specifically concerned with the influence the h-index has on garnering funding from national and international sources.

Both groups indicated the importance of performing research that is relevant for clinicians, focusing on high quality randomized controlled trials and methodological papers to generate interest. The impact metrics and “*number of publications don’t count if they are not transformed into clinical practice*”, one said. The belief that publishing clinical guidelines, methodological work and reviews has a positive effect on individual metrics was also prevalent in both groups.

The near equivalence of the publication rate in the two groups is surprising, yet as the Low *a* researchers tend to have a lower total number of co-authors on their papers, it is likely they take on a larger bulk of the work in bringing each article from concept to publication.

For further clarity, in Table 4 we summarize the outcome of the comparison between the publication strategies and the actual publication outcomes of High and Low *a* researchers.

**Table 4:** Confirmation of publication variable preference in the examined high *a* and low *a* research groups.

Researcher group	Variable	Preference confirmed by actual publication metrics
Low *a*	field specific journal importance	Yes
	local collaboration importance	Yes
	h-index, *h*, importance	No–preference inverted
	number of publications, *N*_*P*,_ importance	No–preference inconclusive
High *a*	number of citations, *N*_*C*,_ importance	Yes
	high JIF importance	Yes
	international collaboration importance	Yes

Due to the open form of our interview process, when asked about their publication strategies several Low *a* researchers elected to speak about news and social media dissemination of their work. Research impact can also be measured by changes in the real world, by affecting policies, services, and health guidelines … Preferring to disseminate knowledge via local media is then a logical, albeit less quantifiable, path. The mindset of wanting to publish research and disseminate it rapidly within the research community pairs well with the turnaround time of lower impact journals, as a general practice, and may be a secondary factor in choosing the appropriate place to publish research. Additionally, research in specialist fields may not have broad enough appeal to fit within the scope of high JIF journals.

All things being equal, strategically choosing a co-author can be a boon. If there are two potential partners to work with, researchers with High *a* tend to choose the one which will help get their work into a journal with a higher JIF. In one example, by including a co-author who is a journal editor or well-renowned researcher experienced at writing for high JIF. Preferring to add a researcher from another institute or country as a co-author can generate broader appeal and potentially more citations. Researchers with a history in publishing in high JIF are probably also engaged in interesting and unique projects. Our work suggests that researchers with High *a* tend to wisely select their collaborators and research projects, and this eventually pays off leading to a higher citation count overall.

The degree of efficiency, *a*, is by no means a target for researchers or analysts to judge the impact of a researchers’ output. It could be used as a tool to help researcher support services better identify which researcher can use help in what area.

We note that a limitation of this work is that we have only interviewed 9 individuals, within a single research unit of clinical research. We are also aware that the MCA method becomes more valid with a larger pool of subjects compared to the investigated variables. As all the participants in our study are senior faculty, it is impossible to separate the impact their established position may have on their publication strategies. The choices described above may not be open to every researcher, let alone junior researchers. Additionally, the motivation for the identified publication strategies remains unclear. Whether these researchers choose certain publication strategies due to personal preference, trendiness of their topic, funding opportunities, departmental support or prestige we can not say. Further research needs to be done to uncover the motivations for the identified publication strategies. We are therefore fully aware that our results may not be generalizable to the research community as a whole, yet we have shown indications of different publication strategies employed by researchers who have achieved at least some degree of academic success.

## Conclusions

We identified indications for two styles of publication strategies in use by researchers in the Department of Clinical Research at the University of Southern Denmark. Researchers with Low *a* focus on original research supported by a local network of collaborators and disseminate knowledge through the media and field-specific journals. Researchers with High *a* focus on international collaboration and prefer to invest the required time to publish research in journals at the top tier of impact factor. Through quantitative analysis of their research output paired with interviews of individual researchers, we have indications that the degree of efficiency, *a*, can be used to identify researchers that employ differing publication strategies. Both styles of publication strategy can lead to a successful academic career, and high h-index overall, even though the methods of the High *a* researchers were more effective at moving the h-index in the previous 5 years.

## Supporting information

S1 File (PDF)
