# Supplementary material for: Effective publication strategies in clinical research
Source: PLoS One. 2020 Jan 30;15(1):e0228438. doi: 10.1371/journal.pone.0228438 (PMC6992234; doi:10.1371/journal.pone.0228438)
Supplement: S1 File — (PDF) [file pone.0228438.s001.pdf]

Supporting Information

Effective publication strategies in clinical research

Daniella B. Deutz[*¶], Evgenios Vlachos[¶], Dorte Drongstrup, Bertil F. Dorch, Charlotte Wien

University Library of Southern Denmark, Odense, Denmark

* Corresponding author

E-mail: dbd@bib.sdu.dk (DBD)

¶ These authors contributed equally to this work.

Contents

S1. Publication data of the senior researchers at the Department of Clinical Research 2

S2. Publication data of all researchers at the Department of Clinical Research 5

S3. Interview guide and questions 7

The data presented in this publication is shared at DOI:10.5281/zenodo.2608752, excluding data containing personal information.

S1. Publication data of the senior researchers at the Department of Clinical Research

We selected 18 researchers to interview by extracting the publication data of 75 researchers affiliated with the Department of Clinical Research at the University of Southern Denmark (SDU) in senior faculty. To restrict ourselves to citable publications, we limited our study to Scopus, a commercial publication database, and the publication types that it indexes. We note that some non-negligible percentage of their publications are likely published in languages other than English and are excluded from this analysis. We started with a list of 78 researchers at the Department. Each researcher was matched to an author ID (or ID's) by searching the Scopus author index with their full name and affiliation with SDU and/or the Odense University Hospital (OUH). If the correct affiliation could not be confirmed, the author was excluded from the study. With 75 authors remaining, each confirmed author's publication data was extracted from the Scopus Application Programming Interface (API) via the Python Scopus package, on Jan. 2nd 2018. The output of the Python script was verified by manually checking a random sampling of 20 % of the publication data. The h-index, h, and degree of efficiency, a, were calculated from each author's number of citations per paper N_{Cpaper} and the total number of publications, N_P.

The h-index, h, of all researchers at the Department of Clinical Research is shown in Fig. S1a-c as a function of their number of citations, N_C, number of publications, N_P, and researcher age, RA. The h-indices range from 4 to 77, with an average of h = 32±16, see Table S1. There is an obvious positive correlation between N_C and h (with r = 0.90) and N_P and h (with r = 0.89), summarized in Table S2. After all, if one does not have enough publications or citations the h-index cannot increase. The h-index is also positively correlated with the age of the researcher, RA, although the correlation is less strong (r = 0.42). Differences in citation culture from field to field limit the attainable h-index. Even though the researchers in clinical science are in relatively close fields, the absolute value of the h-index is not necessarily comparable from one subfield to the next.

The relationship of the h-index to the degree of efficiency, a, is presented in Fig. S1d, where $a = N_C/h^2$. Those with high values for a, have a relatively high number of citations for their h-index, while those with low values for a, have e.g. ten citations each for their total of ten publications. The bulk of the researchers (71 %), are clustered between $3 < a \leq 5$, with an average h of 33±16. This is similar

to the spread reported by Hirsch 2005 [22] for physicists. Researchers with $a \leq 3$ (13 %) have an h of 27±11, while the top $a > 5$ (16 %) researchers have an h of 30±18.

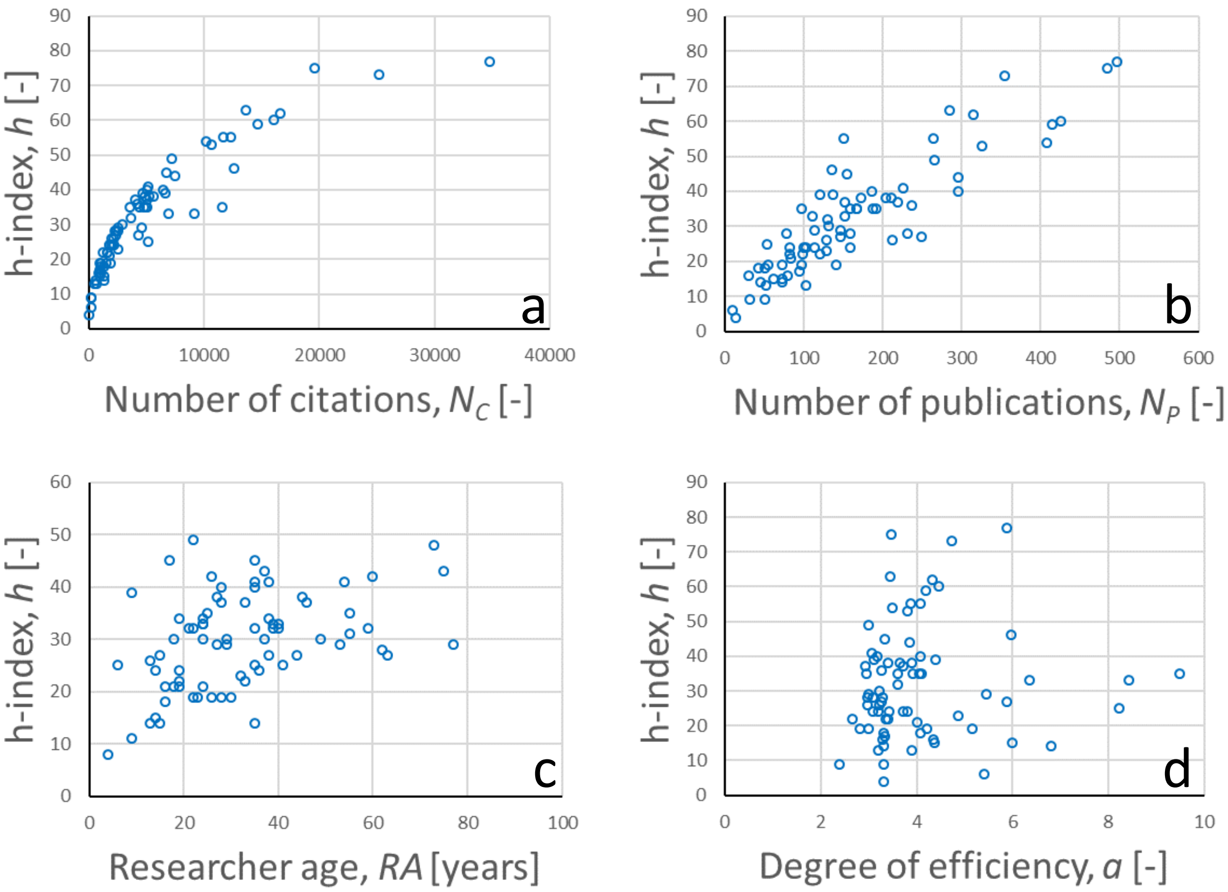

Fig S1. Publication data of researchers affiliated with the Department of Clinical Research at SDU. (a) Number of citations, N_C, versus h-index of the researcher, h. Each point represents a single researcher. (b) Number of publications, N_P, versus h-index of the researcher, h. (c) Number of years since the first publication indexed in Scopus, or Researcher age, RA, versus h-index of the researcher, h. (d) The derived value for the degree of efficiency, a, of each researcher versus their h-index, h.

Table S1. Examination of the h-index of the set of 75 permanent researchers. The researchers at the department are split into 3 groups, depending on the value of the degree of efficiency, a.

	People	Mean Researcher age	Mean h-index	Mode h-index	Median h-index	Min h-index	Max h-index
Department	75	30±9	32±16	35	29	4	77
$a \leq 3$	10	28±8	27±11	19	27	9	49
$3 < a \leq 5$	53	30±9	33±16	24	32	4	75
$a > 5$	12	28±8	30±18	33	28	6	77

Table S2. Correlation between the h-index other metrics for the set of 75 permanent researchers.

		Correlation h - a	Correlation h - N_P	Correlation h - N_C	Correlation h - RA
Department	r	0.07	0.89	0.90	0.42
	variance	0	78	81	18

Table S3. Summary of the publication data of the set of 75 permanent researchers. The researchers at the department are split into 3 groups, depending on the value of the degree of efficiency, *a*.

	Mean h	Mean N_P	Mean RA	Mean N_C	Mean a	N_C / RA	N_P / RA	h / RA
Department	32±16	162±108	30±9	5336±6011	4.0±1.3	165±181	5±5	1.1±0.5
$a \leq 3$	27±11	130±74	28±8	2525±1921	2.9±0.2	165±181	5±5	1.0±0.3
$3 < a \leq 5$	33±16	174±110	30±9	5302±5397	3.7±0.5	167±147	6±6	1.1±0.5
$a > 5$	30±18	139±117	28±8	7829±9019	6.6±1.3	264±264	5±5	1.1±0.6

The mean publication metrics of the three groups are given in Table S3. When we examine the entire department, encompassing 643 people, the same trends hold (See Section S2), and it becomes clear that researchers in the outlying groups (with a ≤ 3 and a > 5) skew younger in their academic career

S2. Publication data of all researchers at the Department of Clinical Research

The publication data of all researchers affiliated with the Department of Clinical Research at the University of Southern Denmark (SDU) was extracted from Scopus via the Scopus API on June 14th 2019. We note that some non-negligible percentage of their publications are likely published in languages other than English and are excluded from this analysis. A list of 791 researchers at the Department were taken from our current research information system (CRIS). pre-matched to a Scopus author ID. The automatic output of the script was verified by manually checking a random sampling of 20 % of the publication data. Researchers with an *h* of 1 and below were excluded from the study.

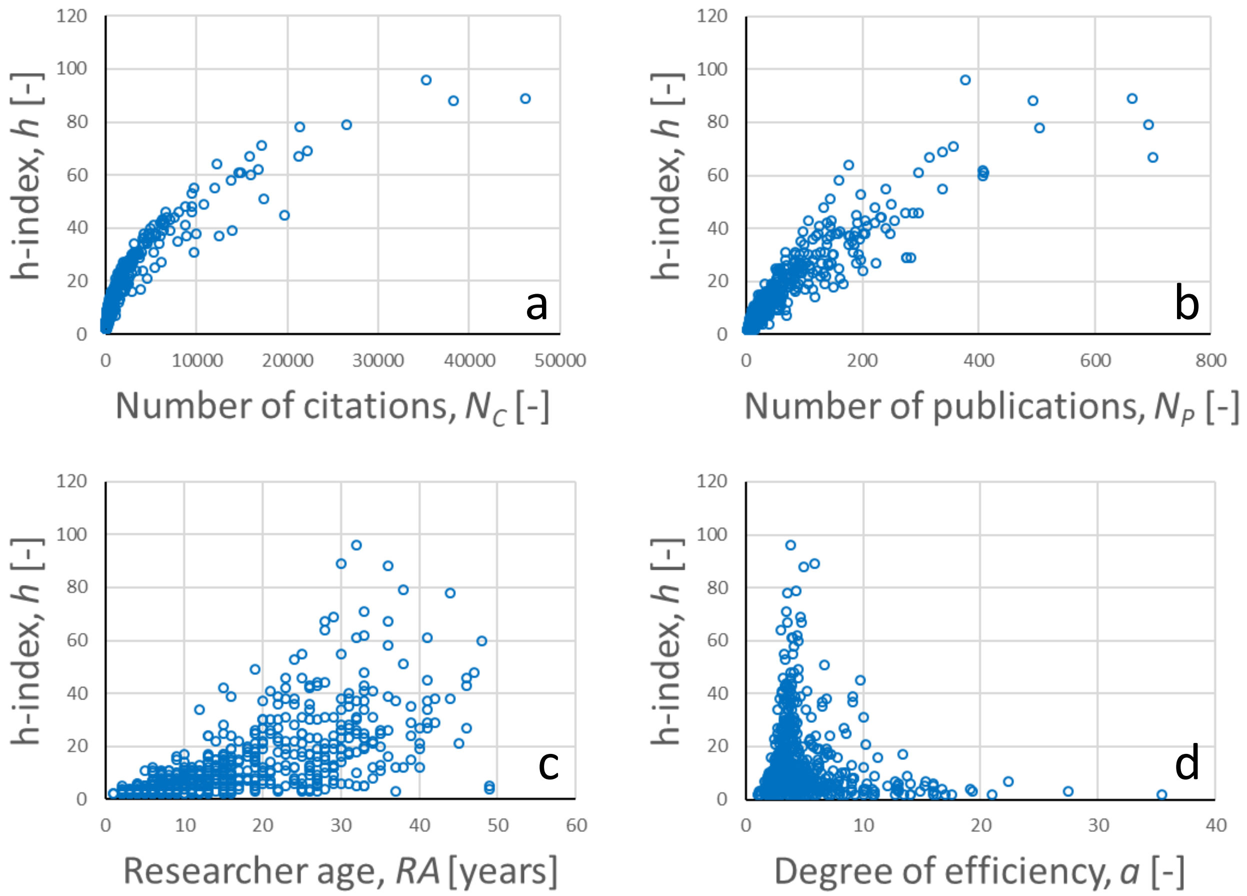

Fig S2. Publication data of all researchers affiliated with the Department of Clinical Research at SDU. (a) Number of citations, N_C, versus h-index of the researcher, *h*. Each point represents a single researcher. (b) Number of publications, N_P, versus h-index of the researcher, *h*. (c) Number of years since the first publication indexed in Scopus. or Researcher age, *RA*, versus h-index of the researcher, *h*. (d) The derived value for the degree of efficiency, *a*, of each researcher versus their h-index, *h*.

Table S4. Examination of the h-index of the Department of Clinical Research at SDU. The researchers at the department are split into 3 groups depending on the value of the degree of efficiency. *a*.

	People	Mean Researcher age	Mean h-index	Mode h-index	Median h-index	Min h-index	Max h-index
Department	645	16 ±11	13 ±14	3	8	2	96
$a \leq 3$	154	11 ±9	8 ±8	2	5	2	64
$3 < a \leq 5$	327	19 ±11	18 ±16	3	9	2	96
$a > 5$	164	16 ±10	10 ±11	2	6	2	89

Table S5. Correlation between the h-index other metrics for all researchers at the Department of Clinical Research at SDU.

		Correlation h - a	Correlation h - N_P	Correlation h - N_C	Correlation h - RA
Department	r	-0.11	0.91	0.88	0.66
	variance	1	83	77	44

Table S6. Summary of the publication data of the Department of Clinical Research at SDU. The researchers at the department are split into 3 groups depending on the value of the degree of efficiency, *a*.

	Mean h	Mean N_P	Mean RA	Mean N_C	Mean a	N_C / RA	N_P / RA	h / RA
Department	13 ±14	53 ±84	16 ±11	1637 ±4131	4.8 ±3.3	65 ±130	3 ±3	0.8 ±0.5
$a \leq 3$	8 ±8	22 ±27	11 ±9	350 ±1106	2.4 ±0.5	19 ±40	2 ±1	0.8 ±0.4
$3 < a \leq 5$	18 ±16	77 ±102	19 ±11	2248 ±4629	3.8 ±0.5	85 ±142	3 ±3	0.9 ±0.5
$a > 5$	10 ±11	34 ±65	16 ±10	1626 ±4577	8.8 ±4.4	68 ±146	2 ±2	0.6 ±0.4

S3. Interview guide and questions

1) Intro presentation of project and bibliometric report.

2) A few words about yourself as a researcher
    a. Senior?
    b. Gender?
    c. Title?
    d. Oriented towards cooperation?
    e. International focus?
    f. Number of Ph.D.-students

3) Can you say a few words about your own publication strategy?
    a. E.g.. what are your considerations before submitting a paper for a journal/conference?
    b. What – in your opinion - is most important: citations or publications and why?

4) We, as a library, conduct research in bibliometrics. Are you aware of any bibliometric performance indicators?

    a. Which measures do you focus on?
        i. Number of citations
        ii. Number of articles
        iii. h-index (Google/Scopus/Web of Science…)
        iv. BFI
        v. Other

5) Awareness of h-index
    a. In your opinion does the h-index play an important role for you and for your publication strategy?
    b. In what ways?

6) Some claim that researchers optimize bibliometric measures – what is your opinion on that?

7) Do you do anything yourself to optimize your bibliometric performance indicators?
